# miR-27a regulates vascular remodeling by targeting endothelial cells' apoptosis and interaction with vascular smooth muscle cells in aortic dissection

**DOI:** 10.7150/thno.35737

**Published:** 2019-10-18

**Authors:** Yudong Sun, Yu Xiao, Huiying Sun, Zhiqing Zhao, Jiang Zhu, Lei Zhang, Jian Dong, Tonglei Han, Qing Jing, Jian Zhou, Zaiping Jing

**Affiliations:** 1 Department of vascular surgery, Changhai Hospital, The Naval Military Medical University, Shanghai, China.; 2 Depaertment of general surgery, Jingling Hospital, Medical School of Nanjing University, Nanjing, China; 3 Institute of Health Sciences, Shanghai Jiao Tong University School of Medicine (SJTUSM) & Shanghai Institutes for Biological Sciences (SIBS), Chinese Academy of Sciences (CAS), Shanghai 200025, China.

**Keywords:** aortic dissection, miR-27a, endothelial cell, apoptosis, FADD

## Abstract

**Rationale:** Aortic dissection (AD) is caused by functional disorder of cells in the aortic wall, which is largely attributed to vascular remodeling. Therapeutic strategies for AD remain limited due to our incomplete understanding of the role of endothelial cells (ECs) in AD pathogenesis. This study aimed to identify the regulatory role of miR-27a in AD and provide a mechanistic basis for a non-invasive treatment of AD.

**Methods:** We harvested aortas from normal and AD patients to explore the expression of miR-27a. *In vitro* and *in vivo* assays were preformed to explore the biological effects of differential expression of miR-27a in ECs and its regulatory effect on AD.

**Results:** MiR-27a was lower in intima of AD samples than in healthy individuals. Downregulation of miR-27a in EC was due to up-regulated expression of fas-associated protein with death domain (FADD) and the activation of apoptosis pathway, which led to apoptosis of ECs. Migration of vascular smooth muscle cells was promoted by EC after downregulation of miR-27a due to enhancement of growth/differentiation factor 8 (GDF8) and repression of matrix metalloproteinase-20 (MMP20) in the co-culture system supernatants. Increase in FADD and apoptosis of ECs to induce AD was shown using mouse models of AD in which miR-27a was stably knocked-down by antagomir. Up-regulation of miR-27a by agomir led to a protective effect on AD.

**Conclusion:** Treatment with miR-27a activator that targets apoptosis of ECs strongly diminished occurrence of AD, providing a new strategy for this disease.

## Introduction

Sudden death due to aortic dissection (AD) can be prevented if individuals at risk are identified and surgically treated 1. Disorders in single genes can be used to identify high-risk individuals 2, however the majority of AD subjects do not have evidence of a single gene mutation. Understanding the mechanisms underlying AD may reveal new therapeutic strategies to prevent this disease and enhance endogenous regenerative mechanisms of aorta. Recent studies have found that microRNAs (miRs) are novel biomarkers and promising therapeutic targets in vascular disease 3-5. One of the two members of the miR-27 family, miR-27a, was found to be involved in various biological processes, such as proliferation, lipid metabolism and drug resistance. It has been confirmed that miR-27a is an endothelial-enriched miRNA that can regulate several functions of endothelial cells (ECs) such as repulsion, angiogenesis and endothelial-mesenchymal transition. 6-8. Although miR-27a plays a regulatory role in functions of ECs in some diseases, the regulatory effect of miR-27a in AD has not yet been explored.

ECs and vascular smooth muscle cells (VSMCs) are the two main cell types in the vasculature 9. AD is defined as separation of the intra-aortic wall with intimal tear for dissection 10. The intima of aorta has a single layer of ECs and the rest of the tissue is composed of collagen and elastin fibers 11. ECs interact and influence the rest of the aorta, including VSMCs. The focus of previous studies related to the pathogenesis of AD has been on VSMCs 12-13. Although some studies have found abnormal appearance of ECs in AD 14-15, the role of ECs in the pathogenesis of AD remains largely unknown. However, endothelial dysfunction is predictive of vascular events and long-term clinical outcome 16-18. Whether dysfunction of ECs is a consequence of AD pathogenesis or can independently drive disease pathogenesis remains unclear.

In this study, we investigated the regulatory role of miR-27a in the beneficial effects associated with function and interaction of ECs with VSMCs after AD by exploring the expression and distribution characteristics of miR-27a in human aorta tissue, identifying the molecular mechanism responsible for the regulatory role of miR-27a on function and interaction of ECs, and examining whether miR-27a can induce beneficial effects in AD.

## Methods

### Tissue samples and ethics statement

The aorta tissues of AD were obtained from ten patients diagnosed with thoracic aortic dissection who underwent the surgery of prosthetic aorta replacement between January 2012 and November 2015 at Changhai Hospital, the Second Military Medical University. Patients with Marfan syndrome, smoking history, Ehlers-Danlos syndrome, aortic aneurysm and other connective tissue disorders were excluded. Control thoracic aorta tissues were taken from ten organ donors who died from nonvascular diseases. All the samples were collected within 30 minutes after aorta excision and rinsed many times to remove the blood and mural thrombus adhering to the vascular wall. After that, these samples were divided into three parts. One part was cut into approximately 2 mm^3^ pieces and placed it in sterile tubes and then froze in liquid nitrogen. The second part was fixed in 4% paraformldehyde and embedded in paraffin. The third part was frozen embedded for in situ hybridization. The clinical information of the subjects in the two groups are presented in Table 1. This study was conducted following the principles outlined in the Declaration of Helsinki and approved by the Changhai Hospital medical ethic committee. All patients and organ donors' direct relatives gave written informed consent.

### RNA isolation and quantitative real-time PCR

Total RNA from the frozen samples or cultured cells was extracted using miRNease Mini Kit (Qiagen, Hilden, Germany) according to the manufacturer's instruction. PrimeScript RT reagent Kit (TaKaRa, Tokyo, Japan) was used to synthesize complementary DNA with500 ng of total RNA. qRT-PCRs were performed using 2μl of resulting cDNA per 20-μl reaction volume containing SYBR green I master. Glyceraldehyde 3-phosphate dehydrogenase (GAPDH) and U6B small nuclear RNA were used as internal control. Controls without template DNA were negative. Real-time PCRs were performed on LightCycler 480 SYBR Green I Master (Roche, Welwyn Garden, Swiss). We incubated the reactions at 95℃ for 30 s followed by 40 cycles at 95℃ for 5s, at 55℃ for 10 s and at 72℃ for 15 s. To verify that the SYBR Green dye detected only one qRT-PCR product, we subjected the samples to the heat dissociation protocol after the final cycle of qRT-PCR to check for the presence of only peak. Mature miRNAs were quantified with specific primers using miScript II RT Kit (Qiagen) for reverse transcription and miScript SYBR Green PCR Kit (Qiagen) for subsequent qRT-PCR as specified by the manufacturer. The primers used in our study are listed in Table 2.

### Fluorescence *in situ* Hybridization (FISH)

LNA-modified probes for miR-27a-3p (5'- and 3'-DIG-labeled), miRNA ISH buffer and Proteinase K were purchased from Exiqon (Vedbaek, Denmark). This experiment was performed on frozen sections following the manufacturer's protocol (Exiqon). Briefly, tissue slides were first warmed and then washed with DEPC-treated 1X PBS 3 times for 5 minutes each, followed by acetylating in 100mM triethanolamine buffer plus 0.25% of acetic anhydride for 10 minutes, permeabilized in PBST (1X PBS plus 0.1% Triton X-100 in DEPC-treated water) for 30 minutes, and washed 3 times for 5 minutes each at RT in 1X PBS. After prehybridization (50% formamide, 10mM Tris-HCl pH8.0, 600mM NaCl, 1X Denhardt's solution, 200μg/mL tRNA, 1mM EDTA, 0.25% SDS, 10% dextran sulfate) at room temperature for 1.5 hours, hybridization was carried out at 56°C overnight in the same hybridization buffer containing 100 nM of miR-27a-3p (5'-GCGGAACTTAGCCACTGTGAA-3') DIG-labeled LNA probes. Slides were sequentially washed with hybridization buffer at 56°C for 15 min. Then hybridization buffer and 2X SSC at the ratio of 1:1, 2X SSC, 0.2X SSC were used to wash slides in sequence at 56°C 3 times for 5 minutes each. MABT buffer (100mM maleic acid, 150mM NaCl, 0.1% tween-20, pH7.5) was then washed at room temperature 2 times for 10 minutes each. Finally slides were then incubated in blocking solution (MABT plus 10% horse serum) for 2 hours at room temperature and then incubated with Anti-Digoxigenin-POD (1:200, Roche) overnight at 4°C. After washing in MABT 7 times for 20 minutes each and 1X PBS 2 times for 10 minutes each at room temperature, signals were developed using Alexa Fluor 633 Dye-conjugated secondary antibody (Roche).

### Western blot analysis

HASMCs or tissues (100mg) was homogenized in 1mL of RIPA buffer containing the protease inhibitor complex (Roche) and phosphatase inhibitors (Roche). Protein concentrations were determined by the bicinchoninic acid (BCA) protein assay kit. All of the proteins were standardized to 1.0 mg/ml. 1X SDS sample buffer was added at the concentration of 25% to the eppendorf tubes. Sonicated for 10-15 seconds and then heated for 5 minutes at 95°C, cooled on ice for 2 minutes and finally centrifuged at 3,000 X g for 1 minute. A total of 20 μl was loaded on a 6% to 12% sodium dodecyl sulfate polyacrylamide gel electrophoresis plate and transferred onto a polyvinylidenedifluoride membrane, so the total amount of each sample of one Western blot gel was 20 μg. Protein concentration was and blocked with 5% bovine serum albumin (BSA) in Tris-buffered saline (10mM Tris, 100 mM NaCl, pH 7.6) with 0.1% Tween-20 (TBST). Primary antibodies were diluted in 5% BSA, and membranes were incubated with an antibody overnight at 4°C.

After washing three times in TBST, membranes were incubated with HRP-linked secondary antibodies (Signalway Antibody) for 2 hours at room temperature. Relative band intensities were evaluated using Image-ProPlus software version 6.0 and β-Tubulin served as an internal control for protein loading. Primary antibodies were FADD (1:1000; Abcam, ab52935 and ab24533), CASPASE-3 (1:500; Cell Signaling Technology, P42574 and 9664S), CASPASE-8 (1:500; Cell Signaling Technology, Q14790 and 4790S), TNF-α (1:500; Cell Signaling Technology, P01375), BCL-2 (1:1000; Abcam, ab32124), BAX (1:1000; Abcam, ab32503), Human Semaphorin 6A (1:1000; R&D, AF1146), β-Tubulin (1:1000; Abcam, ab179513).

### TUNEL assay

Cells were stained by the terminal deoxytransferase-mediated dUTP nick end labeling (TUNEL) technique using an in situ apoptosis detection kit (Roche, Mannheim, Germany) according to the manufacturer's instructions in paraffin embedded sections. Apoptotic cells with red fluorescence were detected with a Pannoramic Confocal Scanner (3DHISTECH, Budapest, Hungary) using scanner software 1.20 Sp1 and Pannoramic Viewer 1.15.4 for Windows. The numbers of TUNEL-positive cells and the media area in human aorta sections were measured in five random microscopic fields.

### Immunofluorescence (IF)

Paraffin sections (5 μm) were blocked by nonimmune serum for one hour at room temperature after being washed in PBS. Endogenous peroxidase activity was inhibited prior to paraffin sections being incubated with primary antibodies (CD31, 1:1000, Abcam) overnight at 4°C. Alexa Fluor 488, 568 and 647 conjugated secondary antibodies (Signalway Antibody, Maryland, USA, 1:200 dilution) were used for the visualization of signals. Semiquantitative analyses of positive signals in samples were performed using Image-ProPlus software version 6.0 (Media Cybernetics, Inc).

### Cell culture and co-culture *in vitro*

Prime HUVECs were purchased from ALLCELLS and cultured in endothelial cell media (ALLCELLS, Emeryville, California) at 37℃ in a humidified atmosphere with 5% CO^2^. HEK 293 T cells were cultured in DMEM (Gibco, New York, NY, USA) supplemented with fetal bovine serum (10%). All the experiments used cells in the third to eighth passages.

The co-culture of HUVECs and HASMCs was performed to evaluate the affection of HUVECs over HASMCs. 1 × 10^5^ HASMCs were planted on a 6-well plant and a 0.4 μm pore size transwell inserts (Corning Costar) were inserted containing 1 × 10^5^ HUVECs. The co-culture system containing both cell types at the same conditions as the monocultures.

### Construction and Infection of miR-27a shRNA lentivirus

The sense and antisense sequences of hsa-miR-27a-3p shRNA were 5'-UUCACAGUGGCUAAGUUCCGC-3', 5'-GCGGAACTTAGCCACTGTGAA-3', and negative control shRNA was 5'-TTCTCCGAACGTGTCACGT-3'. The oligonucleotide sequences were annealed and inserted into the pLKD-CMV-G&PR-U6-shRNA vector (Shanghai Obio Biotech). The lentiviral-based shRNA-expressing vectors were confirmed by DNA sequencing and named pLKD-CMV-GFP-27a. Lentiviruses were generated by transfecting plasmids and packaging vector carriers psPAX2 and pMD2.G with Lipofectamine 3000 into 293T cells at 80% confluence. Supernatants were collected after transfection for 48 hours. For infection, HUVECs were seeded into six-well plates at 60% confluence and infected with the lentiviral constructs (pLKD-CMV-GFP-27a). Green fluorescence protein (GFP) was expressed in the lentivirus; thus, the efficiency of infection was determined by counting GFP-expressing cells under a fluorescence microscope 36~48 hours after infection. Expression of GFP above 90% was considered successful and extraction of RNA and protein was performed.

### Flow Cytometry

The apoptosis of HUVECs was detected by flow cytometry. Cells were suspended in 1X binding buffer (KeyGEN Biotech, Nanjing, China) at a concentration of 1 × 10^6^ cells/ml after being washed twice with DPBS. Annexin V apoptosis detection kit (KeyGen Biotech) was used to stain cells. Dead cells were identified with 7-amino-actinomycin D (7-AAD). Stained cells were sorted using a MACSQuant flow cytometer (Miltenyi Biotec). The figure-displayed dot plots and histograms were obtained using FlowJo software (TreeStar). The experiments were repeated at least three times.

### Antibody microarray analysis

Raybio human apoptosis antibody array (AAH-APO-1) and human cytokine antibody array (AAH-BLG-507) was obtained from RayBiotech. In AAH-APO-1, Each glass slide contains 43 highly specific and well-characterized antibodies in duplicate. These antibodies play important roles in cellular apoptosis. AAH-BLG-507 contains 507 highly specific and well-characterized antibodies and these antibodies play important roles in diverse biological function. Proteins were extracted as described above, biotinylated and hybridized to the microarray. Antibody Microarray Detection Kit (Spring Bioscience, Pleasanton, CA, USA) was used to detect the signal with fluorescent-labeled strepatavidin according to the manufacturer's protocol. Any ≥ 1.2-fold increase or ≤ 0.8-fold decrease in the signal intensity for a single analyst between groups and the fluorescence signal value ≥25 was considered a significant difference in expression.

### Luciferase reporter assay

The miRNA mimics were synthesized from GenePharma. HEK 293T cells were cultured in 24-well plates and co-transfected with 200 ng psiCHECK-2 vector containing 3' UTR of Bcl-2 or 3' UTR of Bid and 40 nM miRNA mimics per well. Lipofectamine 3000 was used in transfections. After 24h transfection, the luciferase analysis was performed with Dual-Luciferase Reporter Assay (Promega, Manheim, Germany) according to the manufacturer's protocol. The relative firefly luciferase activity was obtained after normalizing to renilla luciferase activity.

### Transfection for FADD silencing

A short-interfering RNA (siRNA) specific for FADD and control non-silencing siRNAs were bought from GenePharma (Shanghai, China) (sense: 5'- GCAGCAUUUAACGUCAUAUTTAUAUGACGUUAAAUGCUGCTT-3'). siRNAs were transfected into HUVECs at 40 um using Lipofectamine 3000. After 48h, according to the result of qRT-PCR analysis, the siRNA of FADD suppressed the expression of FADD mRNA by 75% in comparison to the control siRNA.

### Migration and Scratch assays

HUVECs were incubated in 6-well plates in complete EC media to confluence. After serum starvation for 6 hours, a universal blue pipette tip was used to scratch the monolayer of HUVECs and the widths of the scratches in three fields per well were captured. Cells were incubated for 24 h in complete EC media. The same fields were captured again after migration. Differences in the widths of scratches before and after migration were calculated. For transwell assay, HUVECs were trypsinized and cultivated in the transwell chamber (Corning) and cultured in complete medium for 12 hours. Cells migrating to the lower side of the polycarbonate membrane were stained with crystal violet and the average numbers of HUVECs in five random fields per chamber were recorded. All experiments were conducted at least three times.

### Enzyme-linked immunosorbent assay

Cell supernatant levels of GDF8 and MMP20 in the transwell co-culture model were assayed with commercially available quantikine ELISA kits (R&D systems) according to the manufacturer's instructions. Absorbance was detected at 450 nm in a microplate reader.

### Animals

Male mice with FVB background, 3 weeks old, were purchased from the Second Military Medical University Animal Centre (Shanghai, China). All animal procedures were approved by and carried out in accordance with the Experimental Animal Ethics Committee of Second Military Medical University, China, and the Institute of Laboratory Animal Science of China (A5655-01). All procedures conformed to the Directive 2010/63/EU of the European Parliament.

### Aortic dissection murine model

To induce aortic dissection/intramural haematoma, male FVB mice were housed in a temperature-controlled (23 ± 2 °C) room with 50 ± 5 % relative humidity on a 12 hours light-dark cycle (lights on at 7:00 A.M.) and allowed free access to food and water. All the animals were fed with a normal diet and water and administered freshly prepared with beta-aminopropionitrile (1g/kg/day) to 7 weeks old and body weights were measured weekly, as described previously^12^. At 6 weeks old, the animals were diveded into blank group (n=15), model group (n=15), agomiR-27a group (n=15), agomiR-NC group (n=15), antagomiR-27a group (n=15), antagomiR-NC group (n=15). A 200 μl solution of agomiR-27a (15 OD), agomiR-NC (15 OD), antagomiR-27a (15 OD), antagomiR-NC (15 OD) was respectively injected through tail vein of the mice in the groups except model group via a 30-gauge needle. Table 2 listed the sequences of the antagomiRs and agomiRs. At 7 weeks old, manipulated mice except blank group were implanted a 100 μl osmotic pump (model Alzet 1003D; Durect Corp., Cupertino, CA), filled with a solution of angiotensin II (1μg/kg/min) (Sigma Aldrich, St. Louis, USA) for 24 hours. At the endpoints of the experiments, the mice were anaesthetized by phenobarbital (40 mg/kg, respectively), and the sacrifice was resolved following the tissue collection. All mice died before expected end time of the experiment were autopsied immediately.

### Histological analysis

The murine aortas harvested from the ascending aorta to the iliac artery were carefully excised, fixed in formalin, embedded in paraffin, and cut into 5-µm-thick sections. Sections were stained with hematoxylin and eosin (HE) to assess gross morphology. Adjacent sections were used for additional stained with Elastica van Gieson (EVG, for elastic fibers measurement), Masson (for collagen measurement) following standard procedures and examined under light microscopy. Quantification of trichrome staining was performed using Image-Pro Plus software.

### Statistical Analysis

All data were expressed as mean ± SEM. Comparison between two groups was subjected to 2-tailed, unpaired Student's t test. Comparisons of more than two groups used ANOVA followed by Tukey post-hot tests. All the results were from at least three independent experiments. All statistical analyses were performed using Empower (R) (www.empowerstats.com, X&Y solutions, inc., Boston, MA, USA) and R (http://www.R-project.org). P < 0.05 was considered significant.

## Results

### MiRNA expression profiling and in situ analysis reveal miR-27a downregulation and intima localization during AD

**Figure 1:**
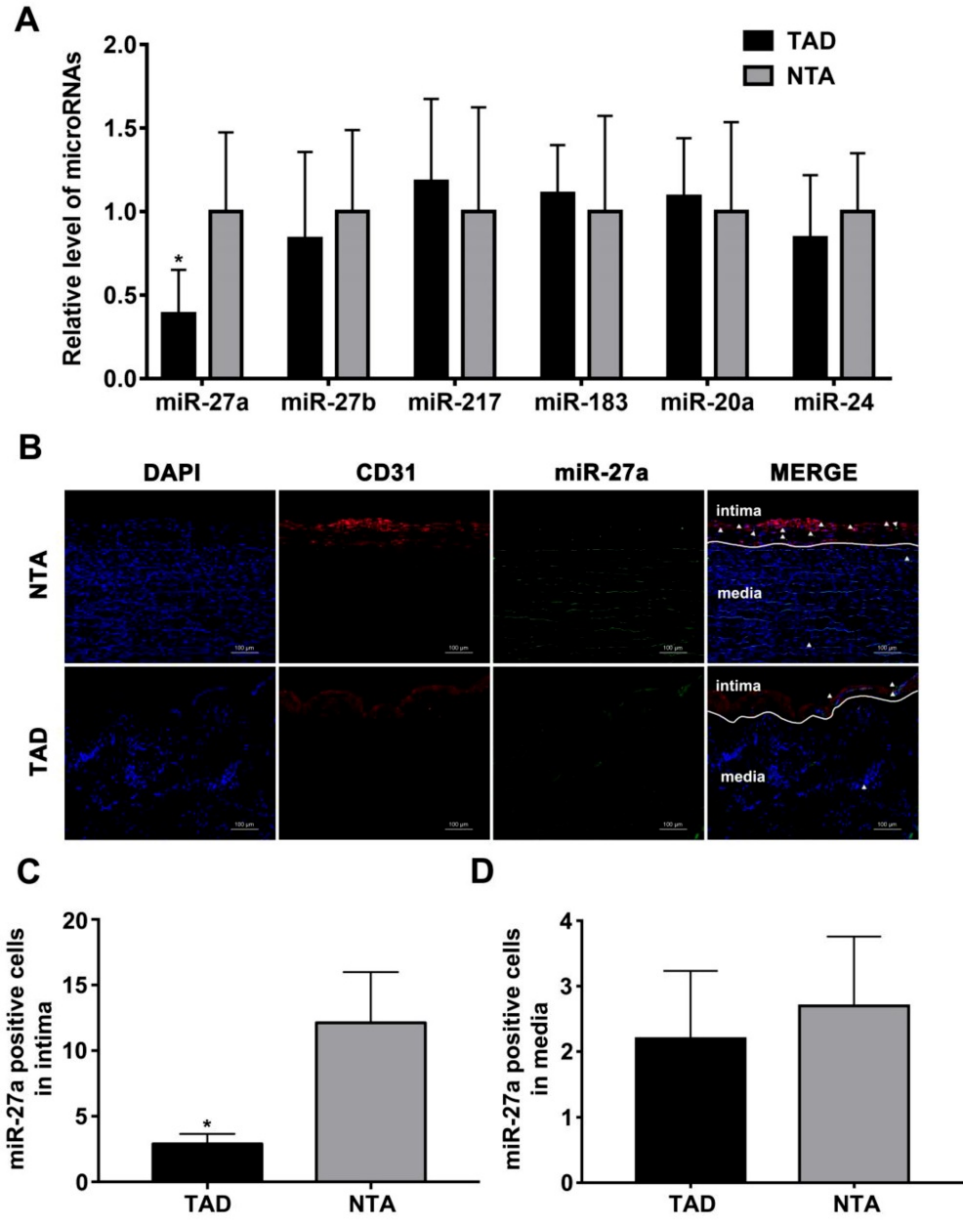
** miR-27a is reduced in intima of human aorta samples with aortic dissection.** (A) Relative quantitation for miR-27a in human aorta samples. (B) In situ hybridization on the same sections of human aorta with miR-27a probe (green signal) and DAPI (blue signal), CD31 (red signal) staining. (C) Quantification of miR-27a positive cell number in intima. (D) Quantitative analysis results of numbers of miR-27a positive cells in media. n=10; Data are represented as mean ± SD; *P<0.05.

We first sought to test whether the intima related miRNA expression differs in human aorta with AD, based on our previously published miRNA profile 19. After statistical testing and filtering for low miRNA expression, six candidates were selected for further analysis according to the fold change in expression after induction of TAD as compared to NTA. As shown in Figure 1A, miR-27a was strongly down-regulated in TAD. To visualize the location of miR-27a in the human aorta, we performed fluorescence in situ hybridization (Figure 1B). The result showed that miR-27a was mainly expressed in aortic intima layer, and was significantly down-regulated in the TAD group when compared with the NTA group (Figure 1C). However, few miR-27a molecules were located in the media of human aorta (Figure 1D).

### MiR-27a exerts an anti-apoptosis function in HUVECs via suppression of FADD

**Figure 2:**
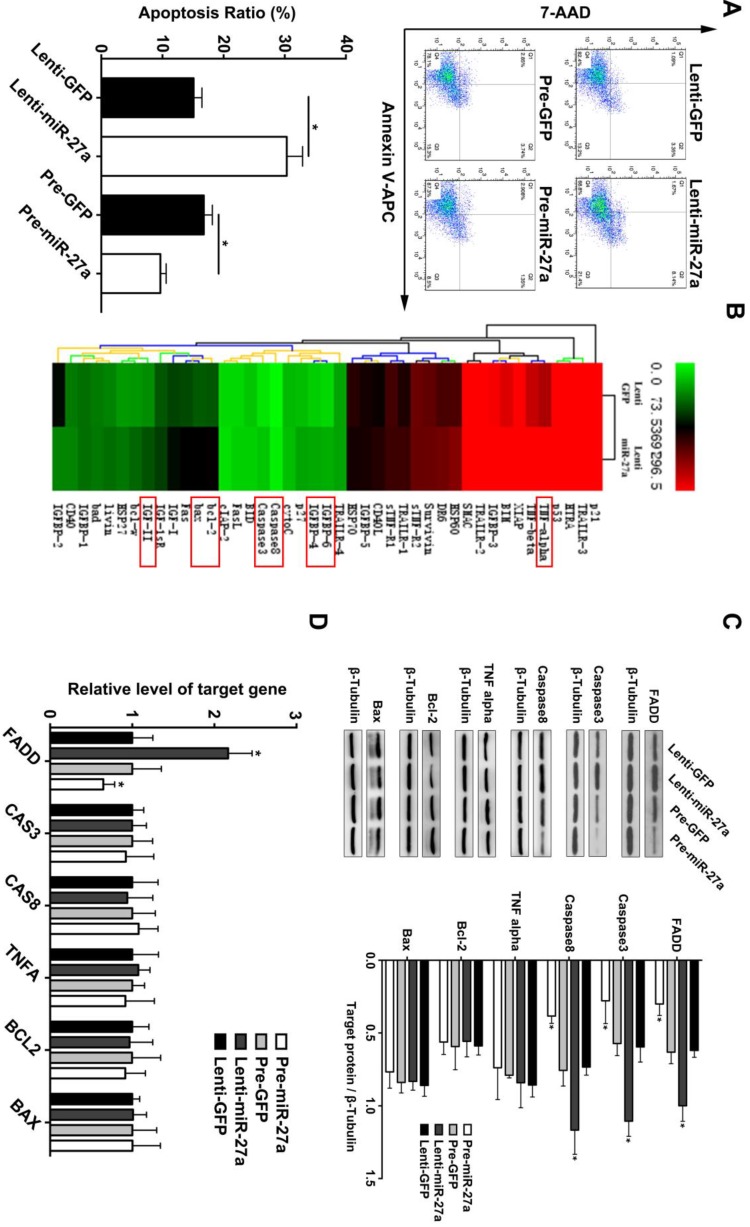
** miR-27a inhibition strongly promotes HUVECs' apoptosis through regulating apoptosis pathway *in vitro* .** (A) HUVECs apoptosis was detected using flow cytometer analysis and the total apoptosis ratio was used for quantitative analysis (n=5). (B) Protein antibody array to identify the differential expression proteins of apoptosis pathway. (C and D) Western blotting and qRT-PCR analysis were conducted to confirm the expression pattern of proteins involved in the HUVECs apoptosis regulated by miR-27a (n=5); Data are represented as mean ± SD; *P<0.05.

Based on the above findings and previous reports of miR-27a as an endothelial-enriched miRNA, we analyzed its effect on the function of HUVECs. MiR-27a was either overexpressed or inhibited by transfection with specific miR-27a precursor oligonucleotides or antisense-inhibitors which were packaged with lentivirus. We achieved a transfection efficacy of >90% and a significant positive or negative modulation of miRNA levels, respectively (Supplementary Figure 1). MiR-27a inhibition significantly promoted apoptosis, while miR-27a overexpression attenuated apoptosis of HUVECs (Figure 2A). To further explore the underlying mechanism of the regulatory effect of miR-27a, we performed an apoptosis protein chip assay with miR-27a downregulation in HUVECs (Figure 2B). After KEGG pathway enrichment analysis of the different expression proteins (Supplementary Figure 2), Fas-associated death domain protein (FADD) was found to be the key point in the apoptosis pathway. FADD interacts with the death domain of Fas and initiates apoptosis 20. It has been demonstrated that FADD has deleterious primary effects on arteries by promoting apoptosis in vascular, thereby weakening vessel walls 21.To further support the protein chip result, we performed western blot and qRT-PCR experiments and the results showed that FADD was significantly upregulated in the anti-miR-27a group. Moreover, the pre-miR-27a group showed the opposite result (Figure 2C).

**Figure 3:**
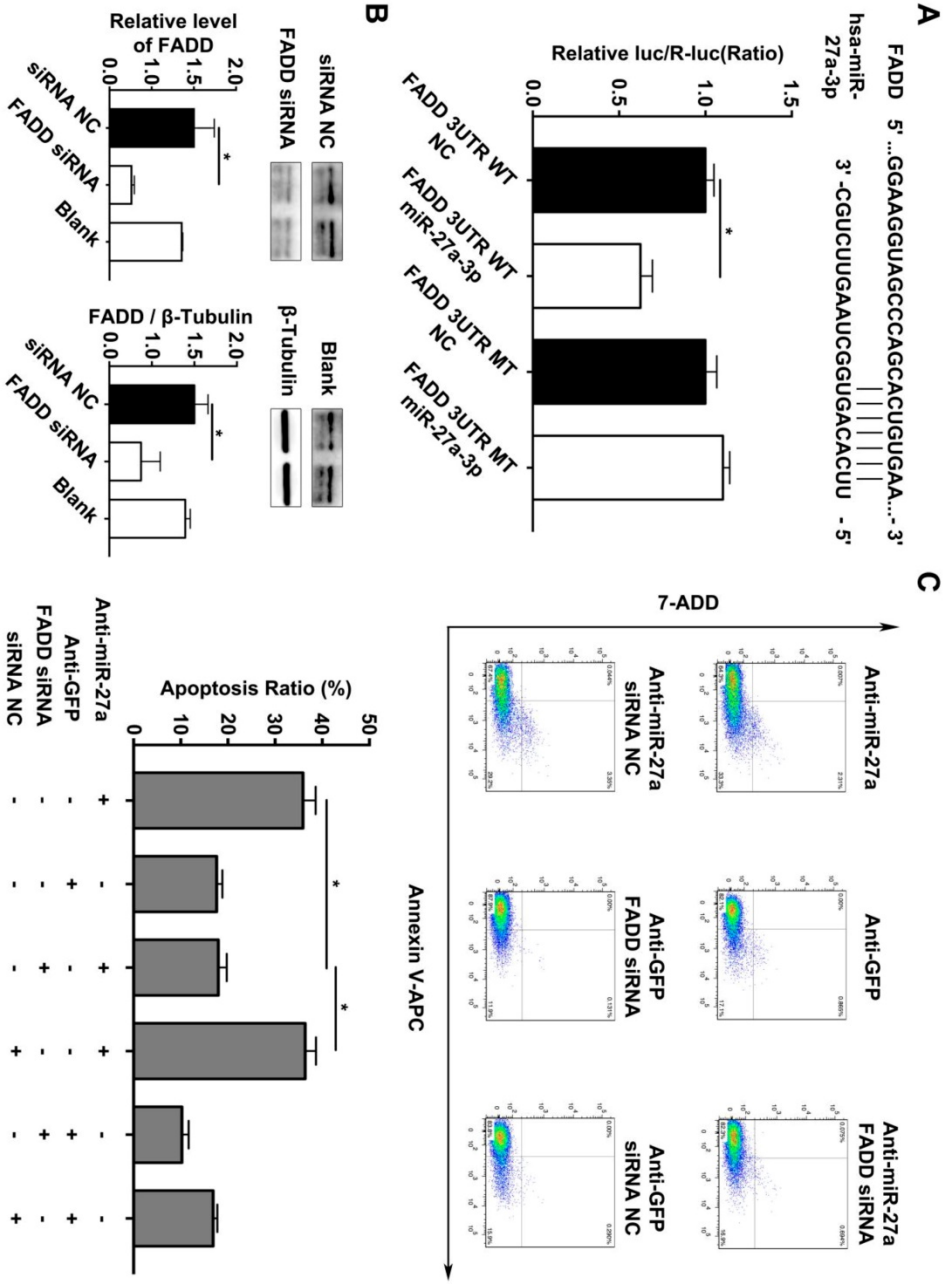
** FADD is a direct target of miR-27a in HEK 293T cells, and apoptosis is partially reduced by FADD inhibition.** (A) Schematic representation of the binding between miR-27a and FADD with mutated sites labeled with black line. HUVECs were transfected with an overexpression plasmid for miR-control (NC) or miR-27a and a plasmid encoding luciferase wild-type (WT) FADD 3'UTR or mutated (MT) FADD 3'UTR lacking the miR-27a-binding site. Luciferase activities were determined 24h later (n=3). (B) qRT-PCR and western blotting analysis on the effect of FADD siRNA. (C) Flow cytometer analysis to evaluate HUEVCs apoptosis cotransfected with lentivirus and FADD siRNA (n=5). Data are represented as mean ± SD; *P<0.05.

In addition, we searched the predicted miR-27a targets in the TargetScan algorithm (www.targetscan.org) and found that FADD might be the target gene of miR-27a. To confirm this finding, a firefly luciferase reporter plasmid fused downstream to segment of the FADD 3'UTR containing either the wild-type putative miR-27a binding sequence or the mutation sequence was generated. We additionally performed luciferase reporter assays to reveal that the 3'UTR of FADD was functional, as evidenced by suppression of the firefly luciferase signal by overexpression of miR-27a but not by overexpression of a control sequence (Figure 3A). To reveal the miR-27a target gene responsible for its effects, we used FADD siRNA to further explain the underlying mechanism of regulatory role of miR-27a in apoptosis of HUVECs. FADD expression levels were modulated by overexpression or inhibition of miR-27a in HUVECs (Figure 3B). The results indicated that the promoting effect on apoptosis of HUVECs could be reversed by inhibiting FADD expression when miR-27a was downregulated (Figure 3C).

### MiR-27a reduces HASMC migration by interfering with expression of GDF8 and MMP20 in HUVECs in a co-culture system

**Figure 4:**
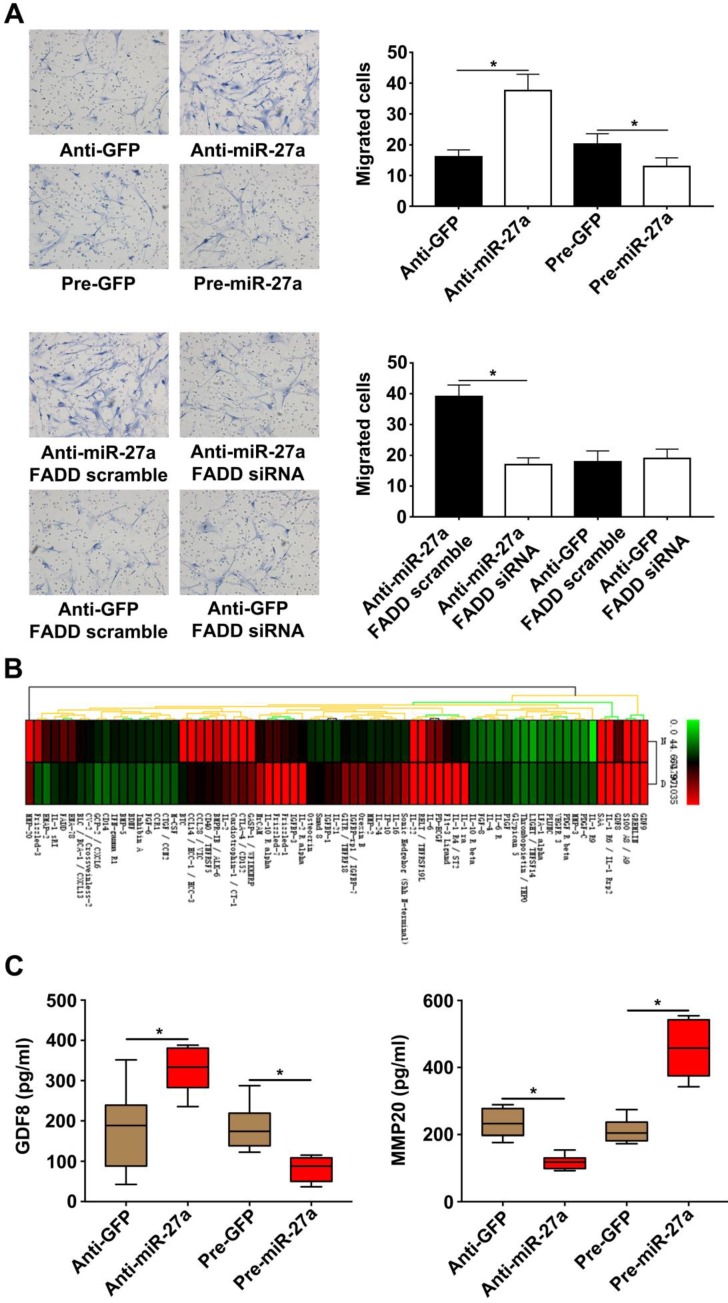
** HASMCs migration was promoted by HUVECs with miR-27a inhibition in co-culture system.** (A) HASMCs migration was detected by crystal violet staining in transwell co-culture system (n=5). (B) Protein antibody array to identify the differential expression proteins in the supernatant of the co-culture system. (C) ELISA experiment for the expression of GDF8 and MMP20 in co-culture system's supernatant (n=5). Data are represented as mean ± SD; *P<0.05.

It has been previously demonstrated that cocultured ECs can regulate VSMC function. VSMC migration is important for AD pathogenesis 22-23. HUVECs transfected with lentivirus were seeded in lower chambers of transwells to verify the role of miR-27a in the migration of HASMCs. The result showed that the miR-27a inhibitor resulted in greater migration of HASMCs. In contrast, up-regulation of miR-27a suppressed migration (Figure 4A). To examine the extent of this modulation by HUVECs, we measured the expression of the proteins in the co-culture system's supernatants which might be linked to mobility of smooth muscle cells with protein antibody array (Figure 4B). Growth/differentiation factor 8 (GDF8) was found to exert major effects on stature, postnatal growth and muscle development which might contribute to vascular smooth muscle contraction and decreased VSMC contraction has been thought as one of the main cause of aortic disease 24. Matrix metalloproteinase‐20 (MMP20) is a member of the matrix metalloproteinase family, a group of zinc‐dependent metallopeptidases, involved in extracellular matrix remodeling 25. The specific characteristics of AD are defined as medial degeneration, fragmentation and degradation of elastic fibers, and remodeling of extracellular matrix in the aorta media 26.According to our antibody microarray analysis result, we chose GDF8 and MMP20 for further research. We additionally performed ELISA with the co-culture system supernatants and found that miR-27a deficiency significantly increased the expression of GDF8 and inhibited the expression of MMP20. Moreover, up-regulation of miR-27a significantly inhibited GDF8 expression and increased MMP20 expression (Figure 4D).

### Site-specific effect of miR-27a deficiency on AD

**Figure 5:**
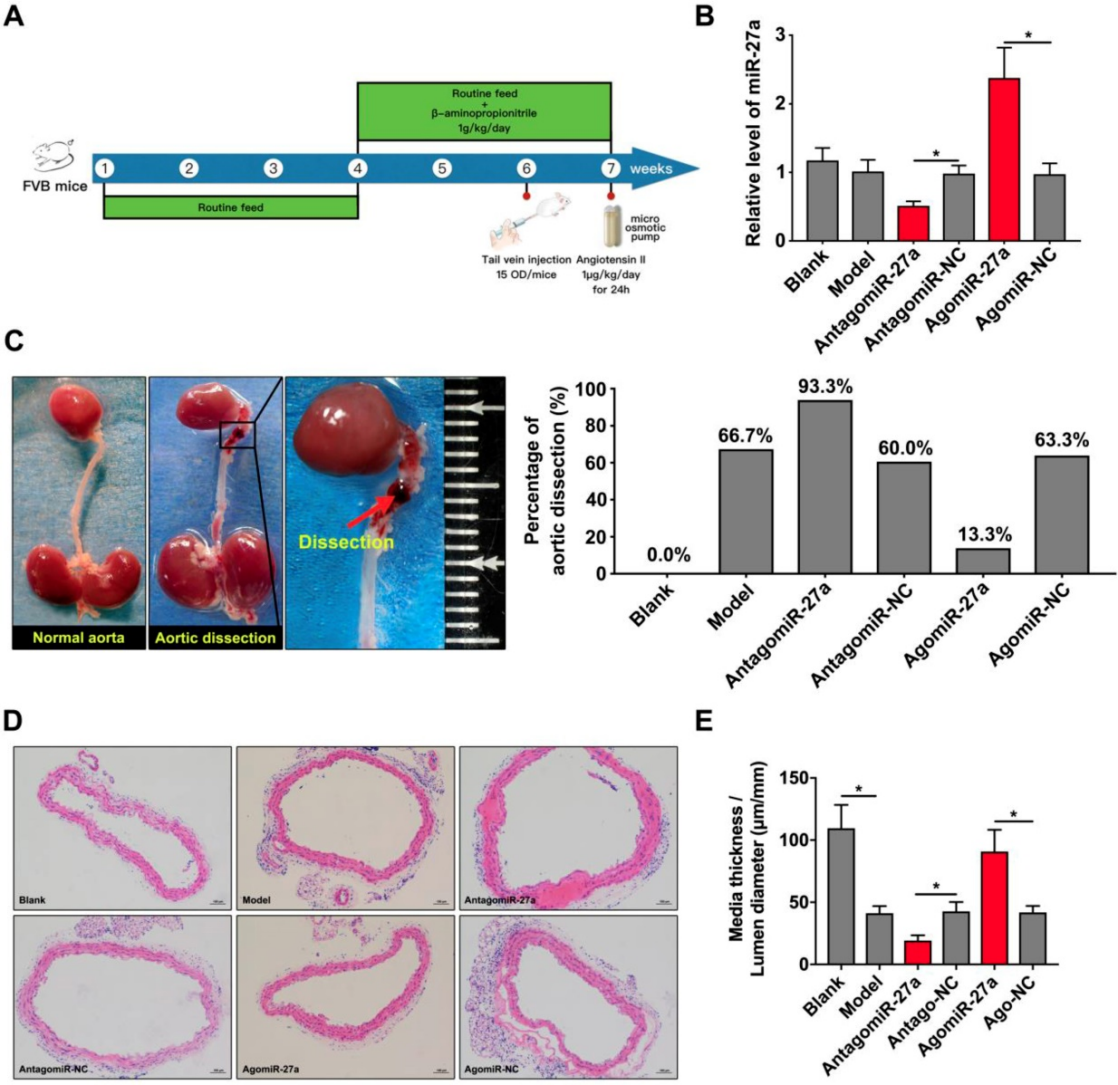
** miR-27a suppression resulted in promotion of AD *in vivo* .** (A) The flow chart of *in vivo* experiment. (B) qRT-PCR experiment to identify the interfering effect on mice aorta of agomiR-27a and antagomiR-27a (n=15 per group). (C) Typical images showed macroscopic features of isolated mice aorta and the incidence rate of AD after treatment. (D) Representative images of H&E staining. (E) Quantification results of media thickness / lumen diameter. Data are represented as mean ± SD; *P<0.05.

To determine the contribution of miR-27a to AD, we crossed male FVB mice with a systemic interference of miR-27a in an AD murine model as described. AntagomiR-27a was effective in targeting miR-27a expression in the aorta leading to a 50% reduction without affecting other members of the miR-27 family. AgomiR-27a significantly increased miR-27a expression to 236% in the aorta (Figure 5B). *In vivo* inhibition of miR-27a increased the incidence rate of AD to 93.3%. However, the incidence rate of AD was decreased to 13.3% when miR-27a expression was increased (Figure 5C). To further identify the morphological feature of aorta, we performed the HE staining to assess the medial thickness (MT), lumen diameter (LD) and the ratio between them (Figure 5D). MiR-27a inhibition strongly reduced MT/LD while upregulation of miR-27a showed the opposite effect (Figure 5E).

### MiR-27a deletion impairs EC apoptosis and regulates GDF8 and MMP20 expression in murine AD model

**Figure 6:**
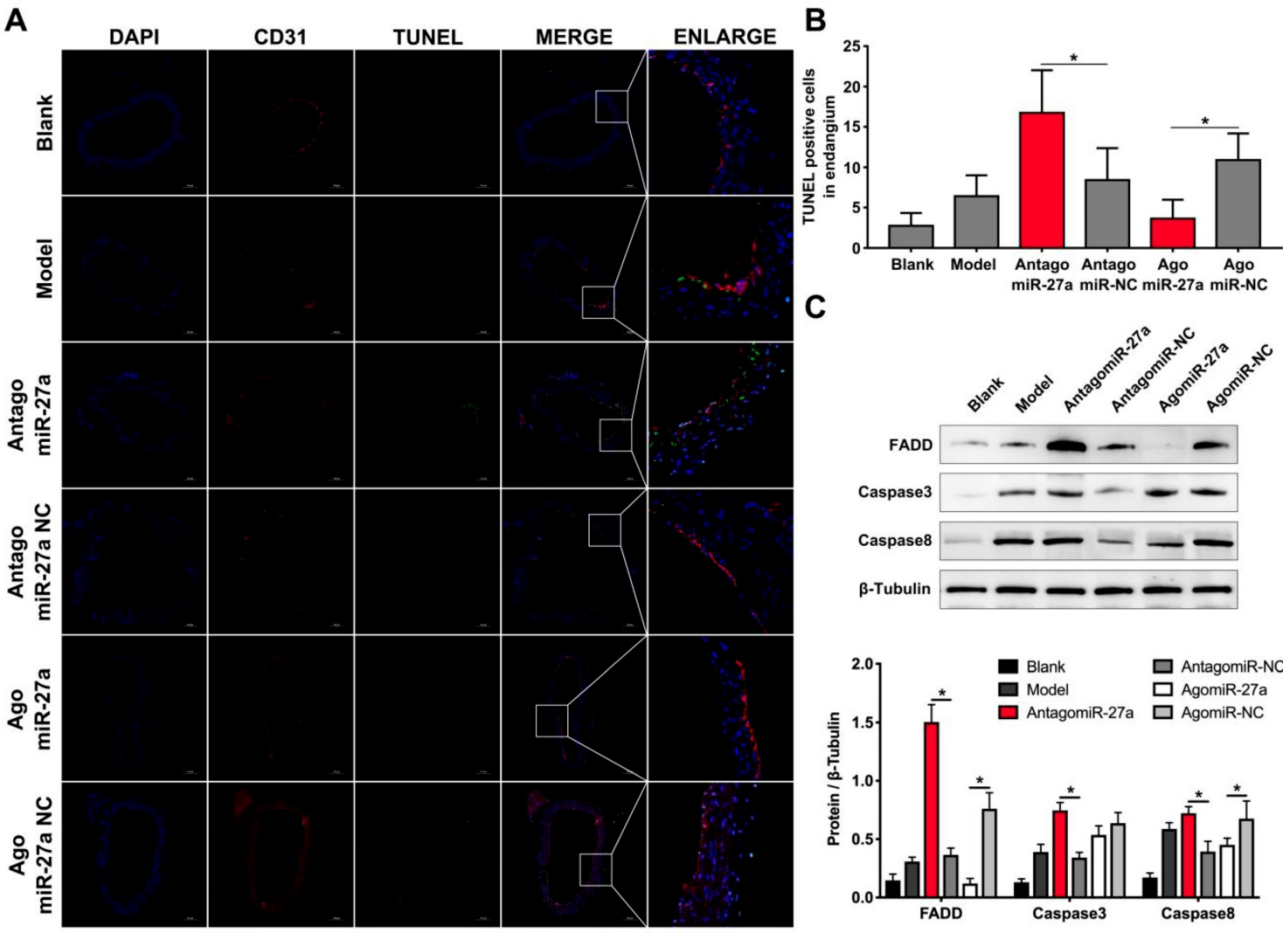
** Inhibition of miR-27a improved endothelial cells apoptosis in AD mice aorta.** (A) Representative images of immunofluorescence staining. Endothelial cells shown in the overlay by a red CD31 staining, TUNEL signal appeared in green and nuclei in blue. (B) Quantitative analysis results of TUNEL positive cells in intima (n=15). (C) Western blotting analysis were performed to confirm the expression pattern of apoptosis related proteins which was identified *in vitro* experiments (n=15). Data are represented as mean ± SD; *P<0.05.

**Figure 7:**
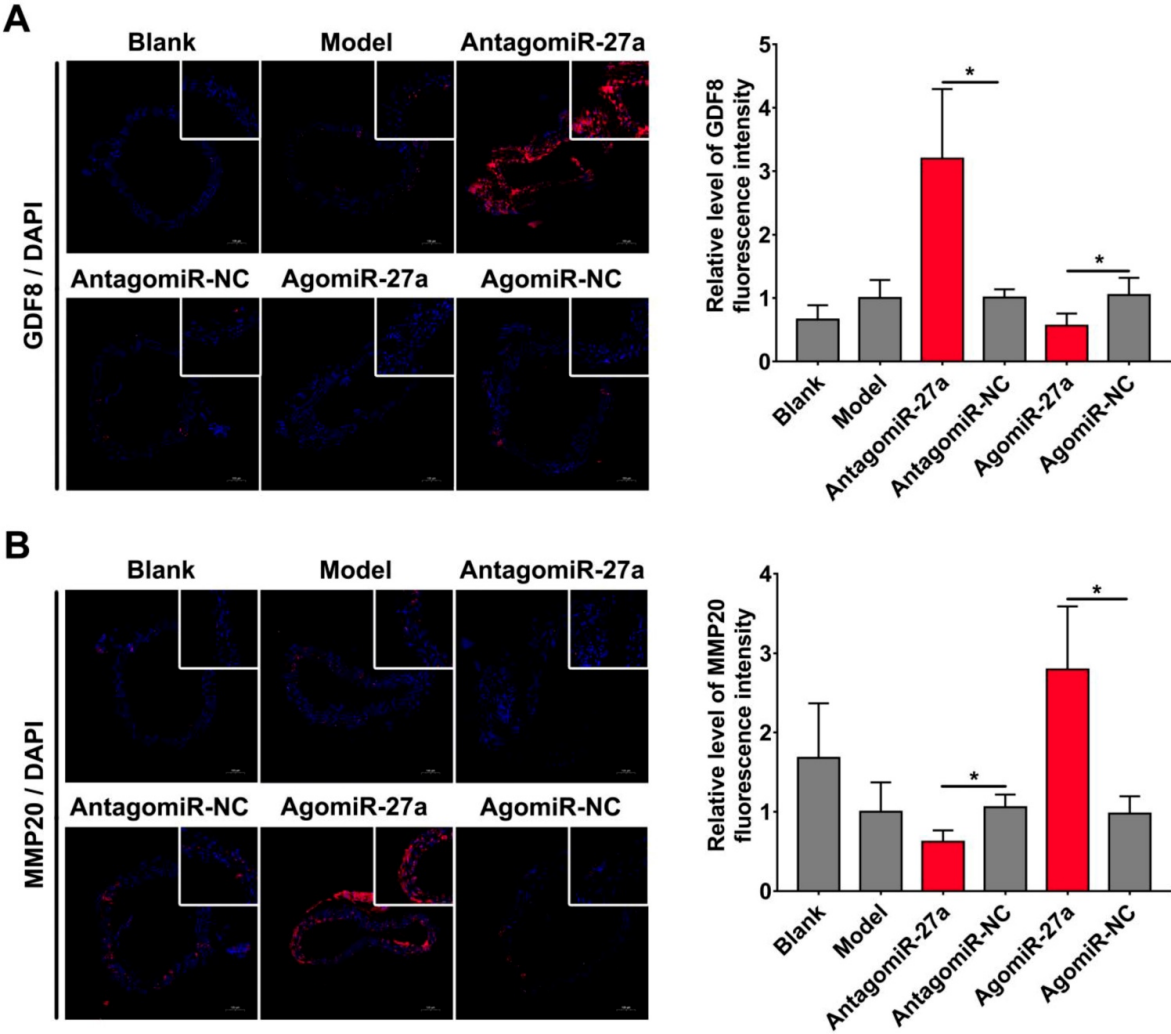
** miR-27a regulated GDF8 and MMP20's expression in murine model for AD.** (A) Immunofluorescence staining and relative fluorescence intensity of GDF8 showing the expression pattern in murine model for AD. (B) Immunofluorescence staining and relative fluorescence intensity of MMP20 showing the expression pattern in murine model for AD. The model group was normalized as one. n=15 per group. Scale bars represent 100μm. Data are represented as mean ± SD; *P<0.05.

To examine whether miR-27a may contribute to the pathogenesis of AD, we explored the effects of miR-27a interference on apoptosis of ECs by TUNEL assay. A significant increase in TUNEL positive cells in intima was observed in mice treated with antagomiR-27a (Figure 6A-B). AntagomiR-27a also induced the expression of apoptosis related proteins including FADD, Caspase-3 and Caspase-8 (Figure 6C). In contrast, agomiR-27a-treated group showed significant decrease in TUNEL positive cells in intima and the expression of these apoptosis related proteins were decreased (Figure 6A-C). Based on the results of the cell co-culture experiment, an immunofluorescence assay was performed to detect GDF8 and MMP20 content in the AD model. Down-regulation of miR-27a resulted in an increase in GDF8 expression and decrease in MMP20, while, up-regulation of miR-27a showed the opposite effect (Figure 7A-B).

## Discussion

Aortic dissection is an important cause of sudden death in previously healthy young persons. A detrimental combination of enhanced aortic cells dysfunctional and exaggerated changes in components of aortic wall causes aorta injury and vascular remodeling. The present study identified decreased miR-27a levels during AD as a deleterious mechanism to induce EC apoptosis, cell interaction, and vascular remodeling in mice. Systemic increase in miR-27a reduced EC apoptosis and prevented the occurrence of AD.

MiR-27a is an abnormal miRNA that is expressed in numerous different human diseases including gastric adenocarcinoma 27, ovarian cancer 28, breast cancer 29 and lower limb ischemia 30. Inhibition of miR-27a inhibits EC sprouting and enhances EC repulsion *in vitro* 6. MiR-27a is involved in pathways essential for endothelial integrity 31. MiR-27a is involved in inhibiting ECs inflammation through regulating nuclear factor κB pathways 32. MiR-27a promotes endothelial-mesenchymal transition and could be a therapeutic target for pulmonary arterial hypertension 8. Furthermore, miR-27a plays a key role in controlling vascular leakiness of ECs by targeting the vascular endothelial cadherin junctional protein 33. Our study showed that deficiency of miR-27a was involved in the regulation of apoptosis pathway by targeting FADD expression in AD. FADD, caspase3 and caspase8 are three key proteins in apoptotic pathway. Our study found that the expression of caspase3 and caspase8 changed in protein levels but not in mRNA levels. We thought this might be due to the activation of apoptotic pathway. Apoptosis is one of the key factors in pathogenesis of AD, and is a potential target for treatment of this disease 34-36. The *in vivo* study showed that miR-27a level was inhibited in the AD model, which was necessary for apoptosis. An inverse relationship between miR-27a and FADD mRNA was found. Together with the protective effects of miR-27a overexpression on the occurrence of AD, these results suggested that the level of FADD must be strictly controlled in AD and miR-27a might be an effective intervention target.

AD, a structural disease in the vascular wall, has become a research hotspot. Previous studies related to the development of this disease were focused on VSMCs 20, 37-39. EC is an important locus of critical regulatory nodes in vascular diseases, and form the interface between circulating blood and the rest of the vessel wall. EC dysfunction within the walls of large arteries is an important contributor to inflammation, barrier dysfunction, blood clotting, hypertension and vascular structural changes 40-45. Various mechanisms induce EC injury and death, which may lead to the detachment of ECs from the vascular wall and thereby cause vascular remodeling 46. Jia et al. reported that endothelial dysfunction plays a role in AD development by endoplasmic reticulum stress dependent microparticles derived from smooth muscle cells 47. EC apoptosis plays significant roles in the focal pathogenesis and development of several vascular diseases, including atherosclerosis, as well as in vascular remodeling. But how EC apoptosis influence AD remains unclear. Apoptosis of ECs is known to be closely related to the enhancement of vascular permeability in AD patients 34,48. Moreover, suppression of EC apoptosis induces inflammation in aorta^47^. ECs dysfunction promotes AD development through pro-inflammatory effect 14. VSMC dysfunction can be stimulated by endothelial injury signals, and participates in the pathogenesis of vascular diseases 49. This study revealed that EC apoptosis was essential for AD in response to suppression of miR-27a. We proposed that ECs in the aortic intima adapted to the enhanced turnover that occurred in response to disturbed flow, and the apoptosis of ECs might trigger AD, which directly induced injury of aortic wall and promoted the instability of vascular structure. Our results showed that suppression of FADD, mediated by the upregulation of miR-27a, negatively regulated EC apoptosis and AD. Thus, this study provided a novel understanding of the role of ECs in the pathogenesis of AD.

A complex process through the formation of stable aorta requires finely orchestrated interactions between endothelial and smooth muscle cells, and the surrounding environment 50-51. To further understand the mechanisms governing the interactions between EC and VSMC, especially the ones that prompt VSMC migration that was shown to participate in the pathogenesis of AD 52, we used a non-contact co-culture model of HUVEC-HASMC. Using this model, we found that the suppression of miR-27a in HUVECs has a greater potential to stimulate HASMC migration through cell communication involving GDF8 and MMP20. It has been reported that increased migration of VSMCs was the main cause of pathological vascular remodeling through undermining the vasculature stability in AD 53. Several miRNAs have been reported to modulate cytokine secretion of ECs by directly or indirectly targeting cellular pathways 54. GDF8 is a member of the transforming growth factor-β (TGF-β) family and it is identified as a strong physiological regulator of muscle differentiation. It has been reported that GDF8 might be involved in the regulation of cells migration 55. Meanwhile, MMP20 promotes cell migration via the Wnt pathway. Thus, we thought the variation of GDF8 and MMP20 in the content of the supernatant might lead to the change of HASMC migration. Understanding the mechanism involved in HUVEC-HASMC interaction and the sequence of events mediating this process are important for the development of vascular therapies and fabrication of vascularized tissues. Consistent with this conclusion, GDF8 and MMP20's expression feature was in accordance with *in vivo* study. These findings suggested that the process of cell communication and the key factors in the process potentially contributed to AD. Further studies are needed to clarify the therapeutic potential of targeting miR-27a for the treatment of AD.

## Conclusions

This study shed light on the important role of miR-27a in ECs during the pathogenesis of AD. A pleiotropic effect of miR-27a was observed in decreasing the incidence of AD, for which EC apoptosis and the interaction between EC and VSMC were risk factors. In line with this, we also observed a significant negative correlation between ECs miR-27a level and FADD, a key protein in the apoptosis pathway. This study provided evidence of how miR-27a in ECs impacts AD, which is associated with endothelial dysfunction.

## Supplementary Material

Supplementary figures and tables.

## Figures and Tables

**Table 1:** The demographic and clinical characteristics of the included TAD patients and controls

	TAD (n=10)	NTA (n=10)
Age, years	49.6 ± 5.8	43.1 ± 8.7
Sex, male:female	10:0	10:0
Hypertension, n(%)	4(40.0%)	2(20.0%)
Hyperlipidemia, n(%)	1(10.0%)	0(0.0%)
Diabetes mellitus, n(%)	1(10.0%)	0(0.0%)
Smoking history, n(%)	5(50.0%)	4(40.0%)
Stanford classification, n(%)		
Type A	10(100.0%)	-
Type B	0(0.0%)	-

**Table 2:** The sequence of primers, angomiRs and antagomiRs in this study.

	Sequence (5' to 3')
**Primers**	
hsa-miR-27a-3p	UUCACAGUGGCUAAGUUCCGC
hsa-miR-27b	UUCACAGUGGCUAAGUUCUGC
hsa-miR-217	UACUGCAUCAGGAACUGAUUGGA
hsa-miR-183	GUGAAUUACCGAAGGGCCAUAA
hsa-miR-20a	ACUGCAUUAUGAGCACUUAAAG
hsa-miR-24	UGGCUCAGUUCAGCAGGAACAG
FADD	GGTGGAGAACTGGGATTTGA
	CAACCATCACTGCCCCTACT
CASPASE-3	AAATGGACCTGTTGACCTGAA
	CACAAAGCGACTGGATGAAC
CASPASE-8	TCACAGCATTAGGGACAGGA
	ACTTTGGGTTTTCCAGCAAG
TNF-α	CATCTATCTGGGAGGGGTCTT
	GAAGTGGTGGTCTTGTTGCTT
BCL-2	CCCGTTTCCTCTGGTGAAC
	GTGTCTCCGTCCTCATCTGC
BAX	ACGACATCAACCGACGCTAT
	GGTGGCAATCTTGGTGAAGT
**antagomiRs and angomiRs**	
mmu-miR-27a-3p angomiR	UUCACAGUGGCUAAGUUCCGC
	GGAACUUAGCCACUGUGAAUU
angomiR NC	UUCUCCGAACGUGUCACGUTT
	ACGUGACACGUUCGGAGAATT
mmu-miR-27a-3p antagomiR	GCGGAACUUAGCCACUGUGAA
antagomiR NC	CAGUACUUUUGUGUAGUACAA

## Abbreviations

AD: aortic dissection
EC: endothelial cell
FADD: fas-associated protein with death domain
GDF8: growth/differentiation factor 8
MMP20: matrix metalloproteinase-20
VSMC: vascular smooth muscle cell
HUVEC: Human umbilical vein endothelial cell
HASMC: Human aortic smooth muscle cell
GAPDH: Glyceraldehyde 3-phosphate dehydrogenase
FISH: Fluorescence in situ hybridization
BSA: bovine serum albumin
TUNEL: Terminal deoxytransferase-mediated dUTP nick end labeling
IF: Immunofluorescence
HE: hematoxylin and eosin
MT: media thickness
LD: lumen diameter
