# Supplementary material for: miR-27a regulates vascular remodeling by targeting endothelial cells' apoptosis and interaction with vascular smooth muscle cells in aortic dissection
Source: Theranostics. 2019 Oct 18;9(25):7961–75. doi: 10.7150/thno.35737 (PMC6831472; doi:10.7150/thno.35737)
Supplement: Supplementary file 1 — Supplementary figures and tables. [file thnov09p7961s1.pdf]

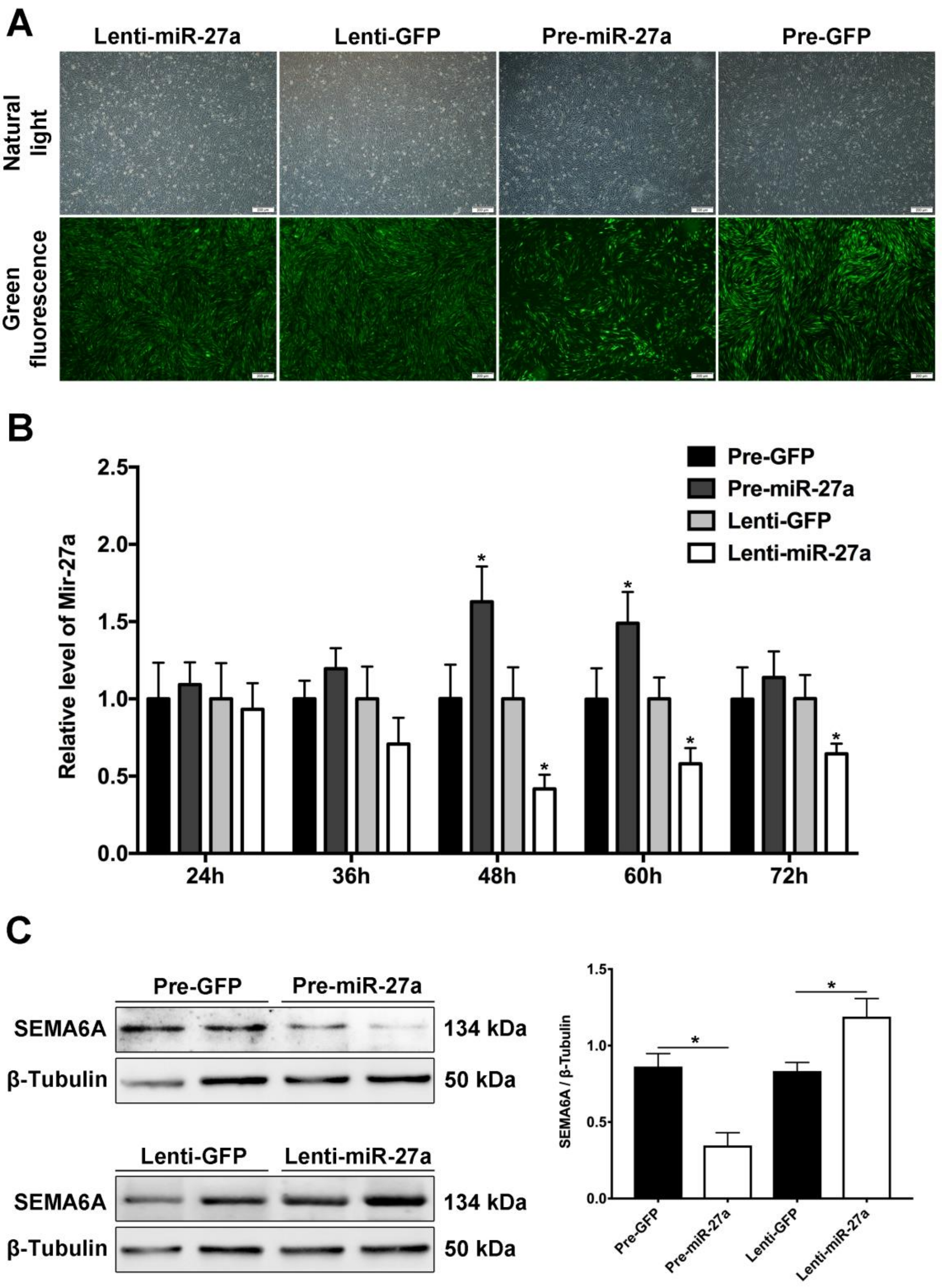
A
Lenti-miR-27a
Lenti-GFP
Pre-miR-27a
Pre-GFP
Natural light
Green fluorescence
B
Pre-GFP
Pre-miR-27a
Lenti-GFP
Lenti-miR-27a
Relative level of Mir-27a
2.5
2.0
1.5
1.0
0.5
0.0
24h
36h
48h
60h
72h
C
Pre-GFP
Pre-miR-27a
SEMA6A
134 kDa
β-Tubulin
50 kDa
Lenti-GFP
Lenti-miR-27a
SEMA6A
134 kDa
β-Tubulin
50 kDa
SEMA6A / β-Tubulin
1.5
1.0
0.5
0.0
Pre-GFP
Pre-miR-27a
Lenti-GFP
Lenti-miR-27a

Supplementary Figure 1. The constructed lentivirus can significantly interfered miR-27a in HUVECs

(A) The MOI test for constructed lentivirus on HUVECs. (B) qRT-PCR result of miR-27a after transfected with lentivirus at different time point. (C) The western blotting of SEMA6A which has been reported as the positive control of miR-27a on HUVECs (n=3). Data are represented as mean ± SD; *$P<0.05$

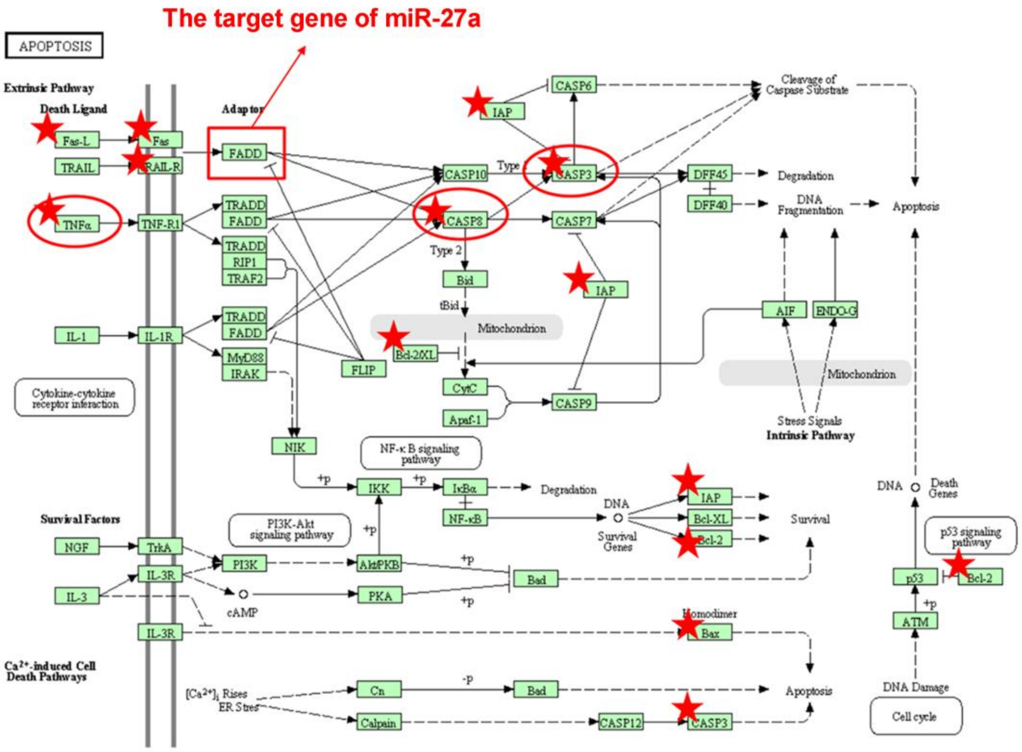

Supplementary Figure 2. The apoptosis pathway image from KEGG
